# Resistance strategy to ageism-based frailty in Italian older women in the COVID-19 pandemic

**DOI:** 10.1371/journal.pgph.0000998

**Published:** 2022-09-14

**Authors:** Ivana Matteucci, Alessandro Porrovecchio

**Affiliations:** 1 Department of Communication Sciences, Humanistic and International Studies University of Urbino Carlo Bo, Urbino, Italy; 2 ULR 7369- URePSSS - Unité de Recherche Pluridisciplinaire Sport Santé Société, Univ. Littoral Côte d’Opale, Univ. Lille, Univ. Artois, Dunkerque, France; Bahauddin Zakariya University, PAKISTAN

## Abstract

The objective of the present study was to examine the relationship between resistance to ageism-based frailty (A-BF) and physical activity (PA) and sport in a cohort of women, aged 65 and older, living in Central Italy. The study was conducted in the spring of 2021 when rigorous COVID-19 containment measures were in force across Italy. A quanti-qualitative investigation was carried out in the cohort. A questionnaire to evaluate older women’s engagement in (PA) and sport was administered and subsequently semi-structured phone interviews with those subjects who were found to be physically active were conducted to evaluate their forms of resistance to the crisis. A total number of 88 subjects responded and participated in the survey. Two tools were used to determine the study outcomes in the quantitative investigation. An altered version of Godin and Shephard’s Leisure-Time Exercise Questionnaire (GSLTPAQ) was used to evaluate the engagement of women in PA and sport. Moreover, subjects’ motivation to exercise was evaluated when they completed the survey using the Behavioral Regulation in Exercise Questionnaire (BREQ-2), a tool that assesses exercise regulation according to the Self-Determination Theory (SDT) framework. The active women resulting from the quantitative investigation were then interviewed in the qualitative investigation, using an interview grid. In the quantitative investigation it was found that PA is correlated with autonomy. Identified and intrinsic regulations prevail in women who are engaged in medium or high PA, vice-versa external regulation, introjected regulation and amotivation prevail in women who are engaged in insufficient PA or who are sedentary. In the qualitative investigation it was found that the participants experienced ambivalence, conflicts and crises at multiple levels (individual, interpersonal and institutional), generating contrasting feelings, which they faced by developing an active, peaceful and silent form of resistance by caring for their bodies and minds engaging in PA and sport.

## Introduction

During the global pandemic declared in March 2020 due to COVID-19—the potentially fatal respiratory virus first reported in Wuhan, China—in addition to the direct impact of the disease, there were also unintended negative consequences on physical and mental health due to public health restrictions, such as reduced health-promoting behaviors (e.g. physical activity and sport) and increased mental duress due to social confinement and health inequalities [1]. Coronavirus disease 2019 (COVID-19) has already been identified as a geriatric health emergency [2]. As older adulthood carries with it an increased risk of having underlying conditions that might increase vulnerability to and impair recovery from COVID-19, distancing from older friends, neighbors, and relatives has been indicated as a necessary precaution to protect older adults. However, an important aspect of the impact of this global pandemic, in addition to the glaring threat of mortality in at-risk older adults, is the pervasive impact of increased loneliness and social isolation [3, 4]. Indeed, loneliness and social distancing are usually associated with poor mental and physical health and are important risk factors for developing the most common mental disorders (e.g. anxiety and depression) [5]. This is known as the “paradox of social distancing” [6] occurring during COVID-19 pandemic.

Women might be found to be frail more often than men. Social frailty is defined as a state of being at risk of losing (or having already lost) resources that are essential for meeting one or more basic social needs [7]. In socio-demographic studies, it appears that the condition of frailty prevails in females, especially in groups with a low level of education and an income below the poverty line [8, 9]. Furthermore, women appear to be disproportionately affected by the negative consequences of COVID-19 restrictions as they make up 70% of health and social-service workers worldwide and are more likely to be in retail and supply jobs [10]. Moreover, more women than men are employed in public and private care for subjects infected by COVID-19 or at risk of being infected [11, 12].

In this paper, frailty was associated with the concept of ageism, referring to older women, while their resistance developed against it was analyzed as related to body care and engagement in PA and sport. The present pandemic also threatens to weave ageism more extensively into the fabric of Western culture [13]. The results of Duncan and Schaller related to people’s feelings of vulnerability to infectious disease [14] suggest that the pandemic itself, as a public health threat, has the potential to increase ageist views. It was found that labeling older adults as an at-risk sub-population inevitably contributed to public and self-stigmatization [15]. It has already been suggested that stereotyping of and disregard for older adults have contributed to the severity of the impact of the COVID-19 pandemic [13].

Sociability [16] as well as non-sedentary lifestyles are usually associated with reduced overall mortality, an increase in life expectancy and a greater likelihood of living an old age in good [17]. But a few is known about the effects of the engagement in physical exercise on the condition of frailty based on age and gender stereotypes. In several studies, gender has been associated with social frailty [18, 19], but there is a lack of consensus in the findings that might stem from the investigated role of (PA) and sport. Engagement in PA is a multi-faceted construct impacted by individual factors, such as motivation and exercise self-efficacy as well as environmental factors, such as social support and recreational opportunity. A reduction in health-promoting behaviors due to preventive public health measures, such as social distancing and the closure of recreation centers, city parks and playgrounds during the pandemic has had an impact on physical activity engagement [20, 21]. Women, in particular, had already reported facing a greater number of barriers to engaging in sport and exercise than men, and these barriers were associated with a lower rate of engagement in (PA) in pre-pandemic studies [22]. Lack of enjoyment and self-consciousness, as well as time constraints are frequently cited by women as barriers to being physically active [23].

This research explored, from a social constructivist perspective, the relationship between resistance to A-BF and PA and sport in a sample of older women living in Central Italy during the implementation of COVID-19 containment measures in the spring of 2021. The study period coincides with the third wave of the spread of the Coronavirus in Italy characterized by the tightening of containment measures (from 6 March to 25 April 2021). With the Prime Ministerial Decree of 2 March 2021, in force from 6 March to 6 April 2021, then extended until 30 April, the new Prime Minister Mario Draghi confirmed the previous containment measures and once again extended the ban on travel between regions. The Decree also introduced a ban on travel for visits to private homes and to reach second homes in areas with high infection rates designated as red zones. All schools were also closed in red zones and in areas where more than 250 infections per 100,000 inhabitants were reported for at least one week. With the Decree law n. 30 of March 13, the yellow zone (low risk) was abrogated as of March 15 and the regions previously designated as yellow became orange (medium risk); during the Easter holidays, a national red zone was also established with the exception of the zones that were already white (lowest risk) for 3, 4 and 5 April.

The paper examines older women’s forms of resistance to A-BF to show the intersection of their experiences with age, PA, sense of identity, and active body perception. The main research questions are: 1. What is the correlation between older women’s engagement in PAand their self-determination? 2. What’s the role of PA in facing A-BF during the COVID-19 pandemic? Drawing on older women’s experiences related to A-BF frames, the study sought to enhance our understanding of the complex interplay of gender, age and the active body in resisting powerful social constructs [24]. The development of effective prevention strategies for the A-BF of older women could reduce its impact at the level of both the individual and the social system, and, consequently, could help to build an age-friendly and a woman-friendly society.

## Materials and methods

### Ethics

#### Ethics statement

Ethical review and approval was not required for the study on human participants in accordance with the local legislation and institutional requirements.

Consent statement:

Written informed consent from the participants was not required to participate in this study in accordance with the national legislation and the institutional requirements.

### Study design

Two investigations were conducted using a mixed method [25] to explore the resistance strategies in older women, aged 65 to 85, living in a region in Central Italy during the COVID-19 pandemic and, specifically, when containment measures were implemented between March and April 2021. This specific timeframe was chosen so that it was possible to focus on participants’ experiences during the epidemic, and in particular, during the implementation of the most rigorous containment measures. The duration of the survey covered the period when the restriction measures were implemented by the Italian government.

### Recruitment

In order to verify the study hypothesis, to ensure that study participants could be recruited rapidly and efficiently in view of the pandemic-related restrictions, as a pilot survey, the case study consisting of a randomly chosen sample based on the telephone directories of a medium-sized town (40,000 inhabitants) located in the central part of Italy was considered. In particular, at the beginning of the phone call it was asked if a woman aged 65 or over who was available to participate in the initiative was present at home.

Paper questionnaires were sent by mail to the older female participants, they were filled out and returned using the same channel. The pre-paid envelope for returning the completed questionnaire was inserted in the envelope. Telephone interviews were then carried out with physically active women. Participants were contacted and recruited for the study by phone. They were provided with information about the study aims and procedures that would be employed, as well as assurances regarding the confidentiality of their participation. The anonymity guarantee was applied later, when the connection between the data in the questionnaire and those of the subsequent interview was made. Once the connection between the questionnaire and the interview has been made, the relevant information containing the names of the respondents was kept by the authors without disclosing any of the microdata, except in the agreed form of the indication of name and age for the purpose of presenting the survey results. Each respondent of the interview was given the possibility that in some cases excerpts from the interview would be cited, indicating only her name and age. This information doesn’t allow the identification of the participants, as their city of residence is unknown. To be able to take part in the investigation participants had to satisfy the following inclusion criteria: be at least aged 65, be free from any impairments. This particular age group of women, aged 65 or older, was targeted so that data on the experiences of people that belonged to the category of older women could be collected.

The target sample size was estimated on the basis of previous methodological studies and the adequacy of the collected data [26] setting it at one hundred units. Once their intention to participate in the study had been confirmed, participants were asked to provide a convenient date and time to be contacted. They were first contacted for the questionnaire that was sent by mail and subsequently for the telephone interview.

### Tools

In the first investigation, data were collected using a questionnaire to evaluate the engagement of the women in PA and sport. Participants were asked to report their current PA levels using the Godin Leisure Time Physical Activity Questionnaire (GLTPAQ) [27]. Amounts of vigorous PA reported in the Godin questionnaire were used to determine if the subjects were physically active. This method has been used to categorize PA in studies including women [28]. Respondents were asked to complete an altered version of Godin and Shephard’s Leisure-Time Exercise Questionnaire (GSLTPAQ) [29]. This 3-item scale asks respondents to indicate the frequency of their engagement in mild, moderate, and strenuous exercise in a typical week. These scores are subsequently weighted by metabolic equivalents of task (MET) (3, 5, and 9, respectively) and summed, yielding an overall weekly physical activity score.

The GSLTPAQ intended scoring is the LSI (Leisure Score Index). The LSI is obtained with the following formula: (frequency of mild × 3) + (frequency of moderate × 5) + (frequency of strenuous × 9). The intended cut-point values for the classification scoring are based on the North American public health PA guidelines. These guidelines are defined as follows: individuals reporting moderate-to-strenuous LSI ≥ 24 are classified as *active*, whereas individuals reporting moderate-to-strenuous LSI ≤ 23 are classified as *insufficiently active* (estimated energy expenditure < 14 Kcal/kg/week) (https://hfjc.library.ubc.ca/index.php/HFJC/article/view/82/49; https://journals.sagepub.com/doi/10.2466/03.27.PMS.120v19x7).

Women were asked the following question: “*In the last two months (March and April 2021)*, *during a typical seven-day period (a week)*, *how many times on average do you do the following kinds of exercise for more than 15 minutes in your free time*?”.

Moreover, subjects’ motivation to exercise was evaluated when they completed the survey using the Behavioral Regulation in Exercise Questionnaire (BREQ-2) [30], a tool that assesses exercise regulation according to the Self-Determination Theory (SDT) [31, 32] framework. The BREQ-2 is an extension of the four subscales of Mullan et al. [33]. The questionnaire has proven to be a valid and reliable tool in both women and men [34] in all the contexts in which it has been employed. The questionnaire measures the following five dimensions: amotivation (e.g. the absence of intent to exercise), external regulation (e.g. exercising because you are told to exercise), introjected regulation (e.g. feelings of guilt when you do not exercise), identified regulation (e.g. appreciating the benefits of exercise), and intrinsic motivation (e.g. exercising because you enjoy it) [20]. For each of these dimensions it is then possible to calculate a relative score. Four items are included in amotivation, external regulation, identified regulation, intrinsic regulation, and three items are included in introjected regulation. Responses were scored on a 5-point scale ranging from 0 = “not true for me” to 4 = “very true for me”.

The relative autonomy index (RAI) can be defined as a single score that is derived from the five subscales of the instrument BREQ-2 (http://exercise-motivation.bangor.ac.uk/breq/brqscore.php, accessed 12 August 2021). RAI yields an index regarding the degree to which respondents feel self-determined. The index is calculated by weighting each subscale and then summing these weighted scores. Specifically, each subscale score is first multiplied by its respective weighting and then these weighted scores are summed together according the following formula: RAI = -3 x amotivation -2 x external regulation -1 x introjected regulation +2 x identified regulation +3 x intrinsic regulation. Higher, positive scores are indicative of greater relative autonomy, whereas lower, negative scores are indicative of more controlled regulation. The data collected from the paper questionnaires were loaded on appropriate statistical software (SPSS) and processed for all research needs.

In the second investigation, in-depth telephone interviews were used with the women found to be active, employing a semi-structured interview grid. Hence, it was possible to fully comply with physical distancing measures in force at the time of data collection. In the social sciences, qualitative semi-structured interviews are one of the most widely used methods for collecting data [35]. Such interviews are particularly effective because they make the exploration of subjective viewpoints possible [36], enabling researchers to collect detailed accounts of subjects’ experiences. They have also been shown to be an excellent method for gauging social responses to the COVID-19 pandemic, providing researchers with a wealth of potentially useful information that could be used in the planning of disease outbreak response measures [37]. After obtaining explicit consent from participants, all the interviews were audio recorded employing a free call recorder smartphone application. The following five main topics with their respective questions to ask interviewees were identified: a) feelings and expectations; a) fears, hopes and resistance; b) risk representation and management; c) body perception and body image; d) difficulty of daily living (mental health, relationship conflicts, and stigma); f) representations of the authorities, media and stakeholders.

The research team was composed of the authors. Both the authors are academic researchers. The telephone interviews were conducted by the female author to solicit older women participation. The authors have experience in conducting interviews, indeed, during their academic career they applied this research tool on several other occasions, as documented in their respective scientific publications. In this investigation interpretive and narrative constructionist approaches to analyze data were adopted. The way women perceived, experienced, and coped with ageing was explored, and their perceptions of the utility of PA and sport for the management of age-related changes in their bodies were examined. Whether participants tended to accept or oppose the social constructs that accompanied older adults in managing the pandemic, if they employed PA and sport as strategies to keep themselves effective and functioning, and if they conceived the active body as a tool to develop PMR were also examined.

### Interviews data analysis

Audio-recordings were transcribed verbatim within one week after each interview. An inductive thematic analysis on semi-structured interview data based on the six-stage comprehensive thematic analysis approach “[38, 39]” was conducted. The analysis involved multiple readings of the transcripts in order to familiarize researchers with the content, the identification of meaningful quotes regardless of their length, labeling them under broader concepts, the organization of such labels around more general themes, and the establishment of relationships between them. Deviant cases were included in the analysis and tables and conceptual maps were created in order to organize the labels and the themes. In the final stage of the analysis process thematic tensions and conflicts experienced by participants were identified and highlighted. The qualitative research software NVivo [40] was used for the analyses of all the interview responses.

## Results—Discussion

### Demographics

The sample originally comprised 100 subjects. A total of 88 older women participated in the study. A number of 22 subjects refused to participate for undeclared personal reasons. The response rate was 88%. Respondents provided demographic and personal information: age, relationship state, employment status, educational level, residential dwelling. The highest percentage of participants were aged 70/74 (n = 38), widowed/divorced/separated (n = 44), retired (n = 37), and had obtained a secondary school degree (n = 30). The mean age of the participants was 73 (SD = 6.0) with a range of 65–85 years of age. See Table 1 for the socio-demographic characteristics of participants (Table 1).

**Table 1:** Participants’ socio-demographic characteristics(n = 88).

**Age**	
**65–69**	**17 (19%)**
**70/74**	**38 (43%)**
**75/79**	**27 (31%)**
**80/85**	**06 (7%)**
**Relationship state**	
**Married**	**28 (32%)**
**Widowed/Divorced/Separated**	**44 (50%)**
**Single/Unmarried**	**16 (18%)**
**Employment status**	
**Full time**	**19 (22%)**
**Part time**	**-**
**Remote work**	**10 (11%)**
**Unemployed**	**-**
**Homemaker**	**22 (25%)**
**Retired**	**37 (42%)**
**Unable to work**	**-**
**Laid off**	**-**
**Educational Level**	
**Elementary or lower**	**22 (25%)**
**Junior high**	**18 (21%)**
**Senior high**	**30 (34%)**
**College or higher**	**18 (20%)**

### Leisure-time physical activity and sport

The GSLTPAQ results indicated that the majority (66%) of the participants were active or moderately active: 25% of the women (n.22) were active; 41% (n.36) were moderately active, and 34% (n.30) were insufficiently active or sedentary.

The study analysis of the differences between the participants revealed that active women generally preferred low- and moderate-difficulty physical activities and/or forms of exercise (in particular, yoga, slow- or fast-paced walking, tennis, ballroom and folk dancing), but several subjects preferred strenuous physical activity/ exercise (in particular, running, jogging and vigorous cycling). This indication, although incomplete, would seem to be not consistent with other research that highlights a female preference for moderate activities and a male preference for high-difficulty activities [41]. Wilson [42] asserted that these differences stem from the physical demands of certain activities, the diverse socializing experiences of males and females and the fact that females mainly stay at home. The study findings are also not consistent with the findings of other studies that show drops in physical activity in older adults, while they are consistent with those showing the engagement of older adults in physical activity and exercise [43, 44] (Table 2).

**Table 2:** Leisure-time exercise.

Godin Scale Score	Categories	Subjects	Average score
**24 units or more**	**Active**	**22 (25%)**	**35**
**14–23 units**	**Moderately active**	**36 (41%)**	**19**
**Less than 14 units**	**Insuf. active/sedentary**	**30 (34%)**	**9**
**M = 19.2 SD = 9.8**			

### Participants’ motivation in sport and physical activity

The internal consistency analysis was obtained by calculating Cronbach’s alpha coefficient. This analysis yielded values between .80 and .90 for the various regulations except for amotivation, which was equal to .54. The analysis of the internal consistency showed that also in our case, apart from the first aspect, the corresponding items of the instrument contributed to define the relative size. Indeed, with the exception of the first aspect, in all the other cases the items we used seemed to define a single aspect, demonstrating that the tool could also be used in this context (Table 3).

**Table 3:** Participant motivation to exercise.

BREQ-2 Regulation	Example questions	α (Cronbach’s alpha coefficient)
**Amotivation**	**I don’t see the point in exercising**	**.54**
**External regulation**	**I exercise because other people say I should**	**.84**
**Introjected regulation**	**I feel guilty when I don’t exercise**	**.80**
**Identified regulation**	**I value the benefits of exercise**	**.90**
**Intrinsic regulation**	**I exercise because it’s fun**	**.87**

### Correlations

PA is correlated with autonomy. Identified and intrinsic regulations (4, 5) prevail in women who are engaged in medium or high physical activity, vice versa external regulation, introjected regulation and amotivation (1, 2, 3) prevail in women who are engaged in insufficient physical activity or who are sedentary. Applying the SDT, it was found that active women’s behavior in PA and exercise was related to autonomous forms of motivation. Indeed, for participants, exercise frequency and intensity showed the strongest correlation with identified regulation and intrinsic regulation. These results are in agreement with the findings of other authors in that the principal types of behavioral regulation of PA and sport in women are identified and intrinsic, whereas introjected regulation has less specific influence [45]. Indeed, we observed that PA and sport in older women tend to show high values for identified regulation of the behavior. On the other hand, the results of our study are not consistent with studies asserting that older women do not consider the activity to be very pleasant (intrinsic regulation) unlike young women [41].

This finding suggests that it is more likely that women will engage in PA and exercise if they feel that exercising is consistent with their identity. This shows that free choice behaviors such as exercise are generally associated with autonomous motivation. Undoubtedly, women who fall into the category of regular exercisers are mindful of the myriad of physiological and psychological benefits associated with routine (i.e. regular frequency) exercise. It is therefore not surprising that regular exercisers aligned their values and goals with routine exercise. It is also easy to understand why women valuing the benefits associated with regular exercise integrated that behavior into their sense of identity. In the present study, it was also observed that subjects who spent the least time exercising were the most amotivated. On all three levels of physical activity there is a direct relationship with the relative motivation, however the strength of this relationship tends to become less important when the level of activity is lowered. Motivation is present in those who do PA, motivation diminishes when the level of PA is lowered (Table 4).

**Table 4:** Intensity of exercise and relative autonomy.

Categories	Leisure time exercise intensity (mean)	Relative autonomy (RAI) (mean)	r (Pearson’s correlation coefficent)
**Active**	**35**	**+ 70,5**	**0.72**
**Moderately active**	**19**	**+ 52,6**	**0.65**
**Insuf. Active/sedentary**	**09**	**- 14,7**	**0.53**

### Older women’s meanings and representations

**Fig 1:**
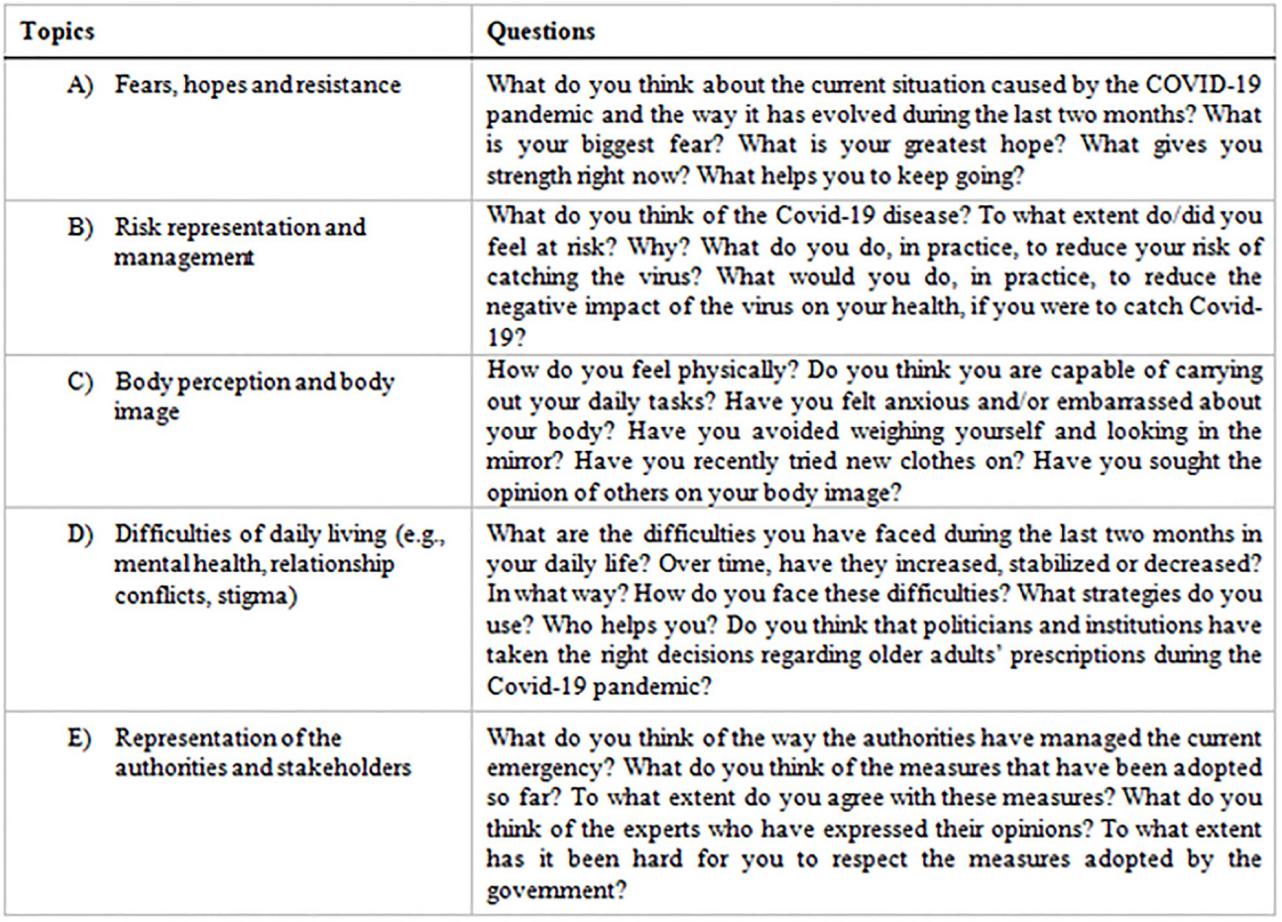
The figure shows the main topics addressed during the interview and the respective questions asked to participants.

In the interviews, were obtained the following results regarding the five main topics addressed: a) fears, hopes and resistance; b) risk representation and management; c) body perception and body image; d) difficulty of daily living (mental health, relationship conflicts, and stigma); e) representations of the authorities, media and stakeholders (Fig 1).

A) Most of the participants stated that both the COVID-19 infection and the measures that were implemented by authorities to contain and mitigate the spread of the virus and the obligation to stay at home had short-term and long-term consequences and risks for their health and wellbeing. As regards the short-term consequences of the pandemic, participants mentioned the risk of coming into contact with the virus and being infected. Among the long-term consequences, they mentioned concern about their mental and social health and the consequences of social distancing and isolation.

*Such a thing had never been seen before*.. *worse than being in war*! *Outside the invisible enemy awaits you*, *inside you fall prey to loneliness*, *melancholy and fear*. *It will take time to fully heal*

*(Maria*, *71 years old)*.

*I think the worst is over*, *yet it’s still as if I didn’t feel completely safe (…)*: *but even though my confidence sometimes wanes*, *my hope for the future never leaves me*

*(Livia*, *67 years old)*.

B) The interviewed older women stated that they felt healthy at the moment, but considered the risk of contracting the virus and believed that it was necessary to respect the measures imposed to avoid the contagion. At the same time, they reported that they were doing things for their physical and mental health, above all, staying active, doing housework and exercise at home and sometimes also using digital platforms to stay physically and socially active.

*Even though I’m not young anymore*, *I feel good and I want to do lots of things*. *Some of my friends let themselves go and become apathetic and lazy*. *I try to keep myself active*: *when I can*, *I go out and go for a walk or even a bike-ride*, *and I also keep busy at home*, *even if I can’t go out*

*(Giovanna*, *69 years old)*.

*I always try not to be negative*! *Yet sometimes when I am alone*, *I get depressed and think about death*: *in this period I think about it much more than before*. *It’s probably because*, *by saying that we are* “*fragile*”, *they make us feel sick and weak*! *I find it hard to identify with this way of looking at older people*

*(Marta*, *67 years old)*.

C) Women also demonstrated interest in their appearance and stated they took care of their body image and the functionality of their bodies. Sport and exercise helped them to achieve these aims.

*As soon as the gym reopened I went back to doing sports*. *In this period I ate a little more than usual when I was alone at home and I felt nervous*. *I also didn’t have the opportunity to exercise*, *so now I want to make up for lost time*!

*(Anna*, *70 years old)*.

*(…) Anyway*, *I don’t stop doing housework and all the chores that keep me busy and keep bad thoughts away*. *I work from home*, *and as soon as I can*, *I go for a run in the park*

*(Teresa 66 years old)*.

D) When asked about social representations of older women, they reported experiencing an identity. They simultaneously felt like “vulnerable victims” who needed to be protected and the “main culprits” for the spread of the virus, those who can expose others to the contagion.

*I saw what happened in the nursing homes (…) «ah but they are so old*, *they have other complications» (…) they may have had complications*, *but if they hadn’t caught the Coronavirus*, *maybe they would still be alive*. *You may as well put us up against the walland shoot us and say «after a certain age*, *we can no longer afford to support you»*

*(Silvia*, *75 years old)*.

*I can no longer invite anyone to my house and my loved ones cannot come*, *but I don’t get it*, *is it a precaution to save us or to save others*?

*(Elly*, *82 years old)*.

E) A lot of women, who did not share the social representations of older adults, reported a sense of having been reduced to a single, at-risk category by the authorities, who did not take into account individual differences, depriving them of their own sense of identity, and thus favoring their stigmatization and victimization in culture and society. They also reported problems with the media regarding information about the spread of the virus and the guidelines for behaviors recommended by authorities, experts and stakeholders. This information was defined as excessive, often conflicting and inconsistent.

*Even the information that comes from television often doesn’t help*, *I have the impression that health is a political issue and a matter of self-interest*. “*It’s every man for himself*”

*(Livia*, *75 years old)*.

*I was able to get a precise idea of the risks of the pandemic and the strategies to counter it*, *but it wasn’t easy*: *it cost me time and effort*. *Experts*, *the media and social networks can help you a lot*, *but you have to be sharp to use them well and garner the best information*, *especially in a crisis situation*

*(Catherin*, *65 years old)*.

It was found that the study participants experienced ambivalence, conflicts and paradoxes crises at multiple levels (individual, interpersonal and institutional), generating contrasting feelings, which they faced by developing an active, peaceful and silent form of resistance by caring for their bodies and engaging in PA and sport. They were afraid of contracting the virus and getting seriously ill, but they also showed a great sense of confidence in their capacity to deal with this adverse situation through their ability to react to achieve individual and social empowerment.

The interviews findings suggest that public health measures specifically targeting the entire over-65 population unintentionally led to an identity crisis among older women. In this identity crisis, older women were caught between being viewed on the one hand, as “vulnerable to” infection, but on the other hand, as a “source of contagion” and a “health risk” for others. The crisis that was brought about by the conflicting feelings of being both “vulnerable objects” and “responsible subjects” of the contagion was exacerbated by contrasting views expressed and adopted by the others: authorities, media, institutions. Participants manifested anxiety over the way others interpreted and responded to the public health measures adopted to contain the spread of COVID-19.

For most of our participants, being associated with the concept of “frailty”, as is mostly the case with aging women, was considered unacceptable, especially because, as we have seen, advanced age is not necessarily defined synonymous with frailty, rather, it is often associated with an active lifestyle, as well as personal development and a sense of fulfillment. Being classified and labeled as “vulnerable” was viewed by the participants as a form of victimization, discrimination and exclusion.

## Conclusions

This research aimed to explore the relationship between resistance to A-BF and PA and sport in a sample of older women residing in Central Italy during the COVID-19 pandemic in the spring of 2021. The survey showed a positive correlation between their engagement in PA and sport and exercise and their autonomy and sense of identity, and demonstrated the role of PA in helping older women in facing the COVID-19 generated crises: crisis of identity, crisis of social status and crisis of physical and mental health.

It was found that older women’s engagement in PA and exercise (even strenuous exercise) was related to autonomous forms of motivation. Indeed, for participants, exercise frequency and intensity showed the strongest correlation with identified regulation (I value the benefits of exercise) and intrinsic regulation (I exercise because it’s fun). This shows that free choice behaviors such as exercise are generally associated with autonomous motivation related to the benefits for the body and mind. The finding suggests that it is more likely that women will engage in PA and exercise if they feel that exercising is consistent with their identity. Motivation is present in those who do PA, motivation decreases when the level of PA is lowered, thus, the more exercise is strenuous the more it can generate autonomy and sense of identity in active older women.

These results of the quantitative investigation are strengthened by the outcomes of the qualitative investigation. In the interviews older women declared they devoted themselves to combat the strong social prejudices related to age and gender, during the COVID-19 pandemic. In the face of this general attitude towards older women, they developed a silent resistance without violating the pandemic-related restrictions. This resistance brought into play the body, generally considered the primary cause of the frailty of older women, and its functioning. In their resistance, they showed that their body was in fact effective and functional in doing PA and sport. The social labeling of older women as weak and vulnerable was therefore countered by the benefits of sport practiced regularly and the pleasure and gratification of engaging in PA. The older female participants were thus helped to cope with the difficulties they encountered on an individual and interpersonal level and became generally able to bolster positive feelings of themselves as a social category capable henceforth of leading the way in finding sustainable solutions and making communities more resistant to crises.

## Supporting information

S1 Text Survey tools. (DOC)
