# Supplementary material for: Resistance strategy to ageism-based frailty in Italian older women in the COVID-19 pandemic
Source: PLOS Glob Public Health. 2022 Sep 14;2(9):e0000998. doi: 10.1371/journal.pgph.0000998 (PMC10021209; doi:10.1371/journal.pgph.0000998)
Supplement: S1 Text — (DOC) [file pgph.0000998.s001.doc]

**QUESTIONARIO SUL TEMPO LIBERO E L’ATTIVITÀ FISICA**

**(****GLTPAQ)**

***IN UN PERIODO TIPICO DI 7 GIORNI (UNA SETTIMANA), QUANTE VOLTE IN MEDIA FAI I SEGUENTI TIPI DI ESERCIZIO FISICO PER PIÙ DI 15 MINUTI DURANTE IL TEMPO LIBERO?***

***(scrivi in ogni riga il numero appropriato)***

Siamo interessati a conoscere quale e quanto esercizio fisico le persone praticano durante la settimana. Usando la tabella sotto riportata, le chiediamo di indicare quante volte la settimana si dedica all’esercizio fisico intenso, moderato e/o leggero. Le ricordiamo che non ci sono risposte giuste o sbagliate e domande ingannevoli. Vogliamo semplicemente conoscere come lei personalmente vive l’esercizio fisico. Le sue risposte saranno mantenute riservate e usate solo per scopi di ricerca.

| **Tipo di esercizio fisico** | **Quante volte per settimana** |
| --- | --- |
| **Esercizio intenso**  **(il cuore batte rapidamente)**  (ad esempio: corsa, jogging, hockey, calcio, squash, pallacanestro, sci di fondo, judo, pattinaggio a rotelle, nuoto vigoroso, cilcismo vigoroso di lunga distanza) |  |
| **Esercizio moderato**  **(non estenuante)**  (ad esempio: camminata veloce, baseball, tennis, cilcismo leggero, pallavolo, badminton, nuoto leggero, sci alpino, danza popolare e folkloristica) |  |
| **Esercizio dolce/leggero**  **(sforzo minimo)**  (ad esempio: yoga, tiro con l’arco arco, pesca in riva al fiume, bowling, rimozione della neve, ferratura di cavalli, golf senza uso di veicolo, camminata facile) |  |
|  |  |

Adapted from Godin, G. (2011).

Translation by Proff. Ivana Matteucci and Alessandro Porrovecchio (2021).

**QUESTIONARIO SULLE MOTIVAZIONI NELL’ESERICIZIO FISICO**

**(BREQ-2)**

***Perche’ ti dedichi all’esercizio fisico?***

Siamo interessati a capire le ragioni delle persone che decidono di pratica e non praticare esercizio fisico. Usando la scala sotto riportata, le chiediamo di indicare quanto è vera ogni affermazione per lei. Le ricordiamo che non ci sono risposte giuste o sbagliate e domande ingannevoli. Vogliamo semplicemente conoscere come lei personalmente vive l’esercizio fisico. Le sue risposte saranno mantenute riservate e usate solo per scopi di ricerca.

|  | **LA SUA MOTIVAZIONE A PRATICARE ESERCIZIO FISICO** | **Per niente vero per me** |  | **Talvolta vero per me** |  | **Molto vero**  **per me** |
| --- | --- | --- | --- | --- | --- | --- |
| 1. | Faccio esercizio fisico perchè altre persone dicono che dovrei farlo | 0 | 1 | 2 | 3 | 4 |
| 2. | Mi sento in colpa quando non faccio esercizio fisico | 0 | 1 | 2 | 3 | 4 |
| 3. | Apprezzo i benefici dell’esercizio fisico | 0 | 1 | 2 | 3 | 4 |
| 4. | Faccio esercizio fisico perché è divertente | 0 | 1 | 2 | 3 | 4 |
| 5. | Non vedo perché dovrei fare esercizio fisico | 0 | 1 | 2 | 3 | 4 |
| 6. | Faccio esercizio fisico perché i miai amici/famiglia/partner mi dicono che dovrei farlo | 0 | 1 | 2 | 3 | 4 |
| 7. | Mi sento in imbarazzo quando perdo un allenamento | 0 | 1 | 2 | 3 | 4 |
| 8. | E’ importante per me fare esercizio fisico regolarmente | 0 | 1 | 2 | 3 | 4 |
| 9. | Non vedo perché dovrei preoccuparmi di fare esercizio fisico | 0 | 1 | 2 | 3 | 4 |
| 10. | Mi piacciono i miei allenamenti | 0 | 1 | 2 | 3 | 4 |
| 11. | Faccio esercizio fisico perché gli altri non saranno contenti di me se non lo faccio | 0 | 1 | 2 | 3 | 4 |
| 12. | Non vedo la ragione di fare esercizio fisico | 0 | 1 | 2 | 3 | 4 |
| 13. | Mi sento incapace quando non faccio esercizio fisico da un pò | 0 | 1 | 2 | 3 | 4 |
| 14. | Penso che sia importante fare lo sforzo di praticare esercizio fisico regolarmente | 0 | 1 | 2 | 3 | 4 |
| 15. | Trovo l’esercizio fisico un’attività piacevole | 0 | 1 | 2 | 3 | 4 |
| 16. | Mi sento “pressato” dagli amici/familiari a praticare esercizio fisico | 0 | 1 | 2 | 3 | 4 |
| 17. | Divento inquieto se non faccio esercizio fisico regolarmente | 0 | 1 | 2 | 3 | 4 |
| 18. | Traggo piacere e soddisfazione dal fare esercizio fisico | 0 | 1 | 2 | 3 | 4 |
| 19. | Penso che l’esercizio fisico sia una perdita di tempo | 0 | 1 | 2 | 3 | 4 |

BREQ-2 (Markland and Tobin, 2004).

Translation by Proff. Luca Pietrantoni and Isaac Ruiz Alfaro (2011).
